# Anxiety disorders in patients with noncardiac chest pain: association with health-related quality of life and chest pain severity

**DOI:** 10.1186/s12955-021-01912-8

**Published:** 2022-01-10

**Authors:** Stéphanie Hamel, Isabelle Denis, Stéphane Turcotte, Richard Fleet, Patrick Archambault, Clermont E. Dionne, Guillaume Foldes-Busque

**Affiliations:** 1 School of Psychology, Université Laval, Pavillon Félix-Antoine-Savard, 2325 rue des Bibliothèques, Quebec, QC G1V 0A6 Canada; 2 Research Centre of the Centre Hospitalier Affilié Universitaire de Lévis of the Centre Intégré de Santé Et de Services Sociaux de Chaudière-Appalaches, 143 Rue Wolfe, Lévis, QC G6V 3Z1 Canada; 3 Centre de Recherche Universitaire Sur Les Jeunes Et Les Familles (CRUJeF), 2915 avenue du Bourg-Royal, Quebec, QC G1C 3S2 Canada; 4 Department of Family and Emergency Medicine, Université Laval, Pavillon Ferdinand-Vandry, 1050 Avenue de la Médecine, Quebec, QC G1V 0A6 Canada; 5 CHU de Quebec Research Centre, Saint-Sacrement Hospital, 1050, Chemin Sainte-Foy, Quebec, QC G1S 4L8 Canada; 6 Quebec Heart and Lung Institute Research Centre, 2725 chemin Sainte-Foy, Quebec, QC G1V 4G5 Canada

**Keywords:** Anxiety, Chest pain severity, Generalized anxiety disorder, Noncardiac chest pain, Panic disorder, Quality of life

## Abstract

**Background:**

Patients with noncardiac chest pain (NCCP) report more severe symptoms and lowered health-related quality of life when they present with comorbid panic disorder (PD). Although generalized anxiety disorder (GAD) is the second most common psychiatric disorder in these patients, its impact on NCCP and health-related quality of life remains understudied. This study describes and prospectively compares patients with NCCP with or without PD or GAD in terms of (1) NCCP severity; and (2) the physical and mental components of health-related quality of life.

**Methods:**

A total of 915 patients with NCCP were consecutively recruited in two emergency departments. The presence of comorbid PD or GAD was assessed at baseline with the Anxiety Disorder Schedule for DSM-IV. NCCP severity at baseline and at the six-month follow-up was assessed with a structured telephone interview, and the patients completed the 12-item Short-Form Health Survey Version 2 (SF-12v2) to assess health-related quality of life at both time points.

**Results:**

Average NCCP severity decreased between baseline and the six-month follow-up (*p* < .001) and was higher in the patients with comorbid PD or GAD (*p* < .001) at both time points compared to those with NCCP only. However, average NCCP severity did not differ between patients with PD and those with GAD (*p* = 0.901). The physical component of quality of life improved over time (*p* = 0.016) and was significantly lower in the subset of patients with PD with or without comorbid GAD compared to the other groups (*p* < .001). A significant time x group interaction was found for the mental component of quality of life (*p* = 0.0499). GAD with or without comorbid PD was associated with a lower mental quality of life, and this effect increased at the six-month follow-up.

**Conclusions:**

Comorbid PD or GAD are prospectively associated with increased chest pain severity and lowered health-related quality of life in patients with NCCP. PD appears to be mainly associated with the physical component of quality of life, while GAD has a greater association with the mental component. Knowledge of these differences could help in the management of patients with NCCP and these comorbidities.

**Supplementary Information:**

The online version contains supplementary material available at 10.1186/s12955-021-01912-8.

## Background

Chest pain is a frequent cause of visits in medical emergency and cardiology settings [1–4]. In approximately 50% of cases, patients present with noncardiac chest pain (NCCP), that is, chest pain in the absence of identifiable cardiac etiology [4–9]. Even though NCCP is often medically benign, its negative impact on quality of life is long-lasting and comparable to that of cardiac disease [10–15].

NCCP is associated with a high prevalence of psychiatric comorbidity (41–88%) [12, 16–21]. The two most common psychiatric disorders in patients with NCCP visiting an emergency department are panic disorder (PD; 14–50%) and generalized anxiety disorder (GAD; 6–33%) [19, 21–27]. These psychiatric comorbidities are associated with a less favourable NCCP presentation and have a serious impact on the patient’s quality of life [21, 24, 28].

Indeed, in patients with NCCP, PD is associated with increased NCCP frequency and severity, increased risk of NCCP recurrence [21, 28–31] and lowered health-related quality of life [28–30]. However, only the physical component of health-related quality of life of the 12-item Short-Form Health Survey Version 2 (SF-12v2) appears to be significantly affected in patients with NCCP who present with comorbid PD [28]. However, these results need to be interpreted with caution, as some are from cross-sectional studies and have yet to be confirmed prospectively [21, 30].

The impact of comorbid GAD on NCCP and health-related quality of life has yet to be assessed. However, the presence of at least one psychiatric disorder is associated with elevated pain severity and life interference due to NCCP [21]. Moreover, GAD has been linked to lowered quality of life in primary care patients, especially with regard to emotional health [32–34]. In fact, the quality of life of patients with GAD has also been found to be similar to that of patients with major depressive disorder and chronic medical conditions, such as arthritis [35–37]. Based on these data, it is likely that the presence of comorbid GAD negatively affects the presentation of NCCP and its consequences on health-related quality of life.

Even if PD and GAD are both anxiety disorders, they are independent diagnostic entities that are likely to have a differential impact on NCCP severity and health-related quality of life in patients with NCCP. Currently, it remains unclear how patients with NCCP and PD compare to patients with NCCP and GAD in terms of patterns of symptoms and health-related quality of life. Therefore, the objectives of the present study were to describe and prospectively compare patients with NCCP, with or without comorbid PD or GAD, in terms of (1) NCCP severity; and (2) the physical and mental components of health-related quality of life. Assessing each component of health-related quality of life is essential in order to understand how PD and GAD respectively and differentially affect patients and to guide clinical decision-making.

Considering that PD is more specifically characterized by interoceptive fear and hypervigilance than GAD [38, 39], it was expected that patients with PD would report greater NCCP severity than those with GAD. Based on the literature, it was also hypothesized that patients with PD would report a lower physical quality of life, while patients with GAD would report a lower mental quality of life. Overall, it was expected that patients with NCCP and comorbid PD or GAD would present with higher NCCP severity and lower health-related quality of life than those with NCCP without comorbid PD or GAD [21].

## Methods

### Design and setting

This prospective cohort study was aimed at describing and comparing patients with NCCP with or without comorbid PD or GAD in terms of NCCP severity and health-related quality of life. Recruitment took place between March 2014 and February 2016 in two emergency departments of the Centre intégré de santé et de services sociaux de Chaudière-Appalaches (CISSS-CA; Centre hospitalier affilié universitaire de Lévis and the Centre Paul-Gilbert de Charny). The CISSS-CA ethics board approved the research protocol (CER-1314–022).

### Participants

This study is part of a larger longitudinal research project. All participants corresponding to the eligibility criteria were selected to constitute the sub-sample of this study. To be included in the study, patients had to meet the following criteria: (1) be 18 years old or older; (2) be fluent in English or French; (3) have visited an emergency department for NCCP with a low risk of death or cardiovascular disease, which was defined as chest pain with no objectively identifiable cause (i.e., normal chest radiography, electrocardiogram and serial cardiac enzymes) and a Modified Thrombolysis in Myocardial Infarction score of two or less [40]. The Modified Thrombolysis in Myocardial Infarction score is based on test results (i.e., EKG and cardiac enzymes), age and medical history. The selected cut-score is associated with a 30-day probability of death, acute myocardial infarction, or revascularization of 5.6 per cent or less [41]. Finally, patients had to have completed the Anxiety Disorders Interview Schedule for the Diagnostic and Statistical Manual of Mental Disorders*,* Fourth Edition (ADIS-IV) [42]. The exclusion criteria were as follows: (1) a terminal illness; and (2) a condition that could invalidate the interview (e.g., an objective medical cause explaining the chest pain, a psychotic state or intoxication, an intellectual disability, or a major cognitive impairment). Patients with missing data on the main study variables were also excluded.

### Procedure

Eligible and consenting patients were invited to undergo a telephone interview and to complete a self-report questionnaire on health-related quality of life (by mail or via the Portail intégré d’applications numériques pour ordinateur (PIANO), a secured Web portal [43]) in the month following their initial emergency department visit and again six months later. The interviewers were doctoral students in psychology (n = 12) who had received initial training and subsequent weekly clinical supervision. All the telephone interviews were audio-recorded, and inter-rater reliability of the diagnoses of PD and GAD was assessed on a randomly selected sample of 20% of the recordings using SPSS random number generator.

### Measures

#### Telephone interview

A structured interview was used to collect sociodemographic data. Average NCCP severity was assessed within 30 days of the initial emergency department visit and again six months later using the following question: “On a scale of 0 to 10, where 0 is no pain and 10 is the worst pain imaginable, how would you rate your average pain during the previous six months?”. PD and GAD diagnoses were assessed with the ADIS-IV [42]. This instrument has good psychometric properties, most notably, good inter-rater reliability for PD (*k* = 0.79–0.82) in patients visiting an emergency department [19, 22] and for GAD (*k* = 0.67) [42, 44].

#### Self-report questionnaire

The 12-item Short-Form Health Survey Version 2 (SF-12v2) measures patients’ perception of their health-related quality of life. The instrument enables one to calculate a Physical Component Summary (PCS) score and a Mental Component Summary (MCS) score [45]. The PCS concerns physical functioning, role limitations due to physical problems, bodily pain, and general health perceptions, while the MCS concerns vitality, social functioning, role limitations due to emotional problems, and mental health. Both are norm-based scores with a mean of 50 and a range of 0–100. Higher scores indicate better health-related quality of life [45]. This scoring method permits comparisons with the 36-item Short-Form Health Survey. The SF-12v2’s reliability and validity have been confirmed in a variety of populations [45, 46]. The validated French adaptation was used in the present study [47].

### Statistical analyses

Descriptive statistics were used to describe the sample according to four mutually exclusive groups (patients with NCCP and PD, patients with NCCP and GAD, patients with NCCP and PD and GAD, and patients with NCCP but without PD or GAD). One-way analyses of variance (ANOVAs) were performed to detect differences between the groups according to age, while chi-square tests were used to compare the groups with regard to categorical sociodemographic characteristics. In addition, the participants in the final sample were compared to the patients who did not complete the final evaluation to assess the risk of selection bias. As well, sensitivity analyses were conducted to determine the impact of the missing data.

Descriptive statistics were used to present average NCCP severity and the scores on both components (PCS and MCS) of health-related quality of life at baseline and the six-month follow-up according to the different groups in the study. Generalized linear mixed models were then conducted to assess time and group effect on average NCCP severity and health-related quality of life. The interaction effect between time and group was assessed for both objectives, and multiple comparisons were conducted to identify significant differences. Effect sizes were estimated by calculating partial eta-squares (η_p_^2^). Generalized linear mixed models were adjusted for potential confounding variables (i.e., civil status and family income), which had been identified with backward selection. All the analyses were performed with IBM SPSS Statistics, version 23.0 (IBM, Armonk, NY), and SAS 9.4 (SAS Institute Inc., Cary, NC) for Windows.

## Results

### Description of sample

**Fig. 1:**
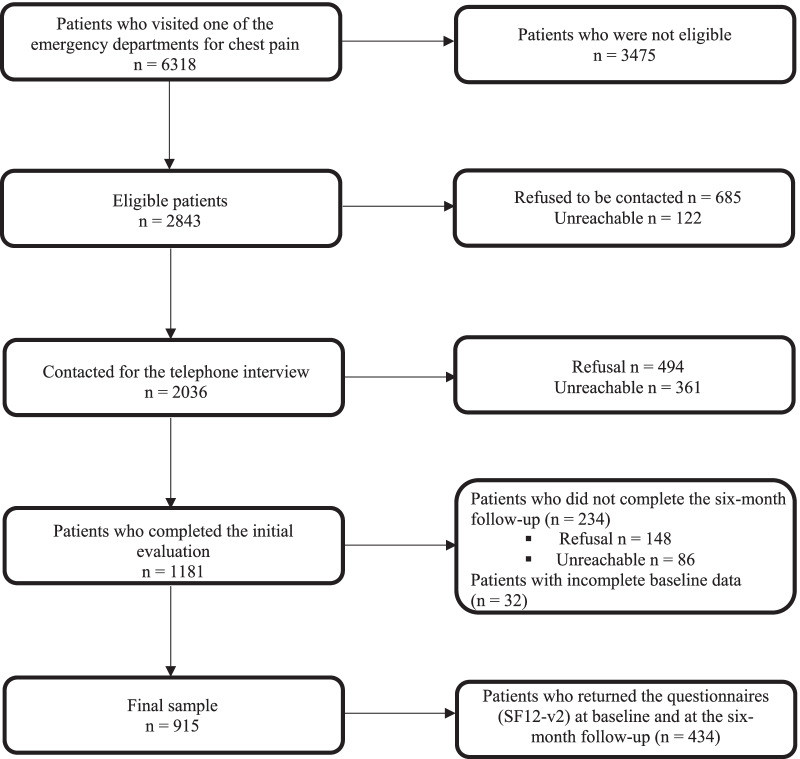
Sample selection process

As shown in Fig. 1, 45% (n = 2843) of the patients who visited the emergency departments for chest pain were eligible for the study, and 71.6% (n = 2036) of them consented to be contacted for the telephone interview. Of these patients, 58% (n = 1181) completed the initial evaluation, and of these, 80.2% (n = 947) completed the six-month follow-up. The final sample consisted of 915 patients with complete data from the telephone interviews (ADIS-IV and average NCCP severity). No statistically significant differences in sociodemographic characteristics were found between the groups (PD, GAD, PD and GAD, no PD or GAD; see Table 1). The patients in the final sample (n = 915) were more likely to have a college or university education (54.0% vs. 37.3%; χ^2^ = 21.623; *p* < 0.001) and to be married/in a common-law relationship (69.1% vs. 61.8%; χ^2^ = 4.790; *p* = 0.032) and less likely to have a low income (58.9% vs. 66.5%; χ^2^ = 5.032; *p* = 0.027) than patients who did not complete the final evaluation (n = 266). Patients in the final sample and patients who did not complete the final evaluation did not differ in terms of average NCCP severity at baseline (*t* (1144) = 0.214; *p* = 0.831). Inter-rater agreement conducted by trained doctoral students in psychology (n = 12) on the ADIS-IV for PD (*k* = 0.78) and GAD (*k* = 0.79) was excellent in the randomly selected sample of 236 (20%) patients.

**Table 1:** Sociodemographic characteristics (n = 915)

	PD (n = 77)	GAD (n = 56)	PD and GAD (n = 48)	No PD or GAD (n = 734)	*t* or chi- square	*p*
Age, mean (SD)	54.1 (16.6)	48.6 (14.4)	52.3 (14.5)	54.2 (15.7)	2.329	0.073
Female, % (n)	41.6 (32)	58.9 (33)	56.3 (27)	50.8 (373)	1.562	0.197
Married or in a common-law relationship, % (n)^a^	63.6 (49)	66.1 (37)	64.6 (31)	70.3 (515)	0.745	0.526
Family income (≤ $59,999), % (n)^b^	68.0 (51)	58.9 (33)	58.7 (27)	55.3 (384)	1.540	0.203
Education (college/university), % (n)^a^	54.5 (42)	57.1 (32)	56.3 (27)	53.6 (393)	0.124	0.946
Employed, % (n)^c^	53.2 (41)	60.7 (34)	54.2 (26)	55.5 (406)	0.269	0.848

PD: Panic disorder; GAD: generalized anxiety disorder; SD: standard deviation

^a^One missing data

^b^34 missing data

^c^Two missing data

A subgroup of 434 patients (47.4%) completed the health-related quality-of-life measure at both time points (SF12-v2). They were significantly older (57.2 vs. 50.6; *t* (913) = −6.527; *p* < 0.001) and less likely to be working (50.0% vs. 60.3%; χ^2^ = 7.617; *p* = 0.006) than those who did not (n = 481). The two groups did not differ in terms of average NCCP severity at baseline (*t* (913) = −0.689; *p* = 0.491) or at the six-month follow-up (*t* (913) = 1.098; *p* = 0.272). However, the patients who completed the health-related quality-of-life measure only at baseline had a lower mental component score than the patients in the final sample (44.99 vs. 48.32; *t* (732) = 4.218; *p* < 0.001).

### NCCP severity

As shown in Table 2, average NCCP severity significantly decreased from baseline to the six-month follow-up (*F* (1, 911) = 176.60; *p* < 0.001; η_p_^2^ = 0.162) for all the patients, regardless of the group. Average NCCP severity also differed significantly according to the group (*F* (3, 911) = 7.29; *p* < 0.001; η_p_^2^ = 0.029). More specifically, average NCCP severity was higher in the patients with PD or GAD than in those without these comorbidities. The patients with PD did not differ from those with GAD (*t* (911) = 0.12; *p* = 0.901). The interaction between time and group was not significant (*F* (3, 911) = 1.50; *p* = 0.212; η_p_^2^ = 0.005). Descriptive statistics on average NCCP severity are provided in Additional file 1: Table S1.

**Table 2:** Panic disorder, generalized anxiety disorder and average NCCP severity (n = 915)

	β (SD)	*p*
*Time effect* 6-month follow-up versus baseline	− 2.59 (0.19)	< .001*
*Group effect*	–	< .001*
PD versus no PD or GAD	0.68 (0.23)	0.003*
GAD versus no PD or GAD	0.64 (0.26)	0.015*
PD and GAD versus no PD or GAD	0.89 (0.28)	0.002*
PD versus GAD	0.04 (0.33)	0.901
PD and GAD versus PD	0.21 (0.35)	0.540
PD and GAD versus GAD	0.25 (0.37)	0.494
*Time* × *group effect*	–	0.212

SD: Standard deviation; PD: panic disorder; GAD: generalized anxiety disorder

^*^p < 0.05

### Health-related quality of life

As shown in Table 3, the PCS scores improved significantly over time for all the patients (*F* (1, 405) = 5.90; *p* = 0.016; η_p_^2^ = 0.014) and differed between the groups (*F* (3, 405) = 7.88; *p* < 0.001; η_p_^2^ = 0.260). More specifically, the PCS scores were significantly lower in both groups of patients with PD than those of the other two groups (GAD and no PD or GAD). The time effect was independent of the group effect (*F* (3, 405) = 0.65; *p* = 0.585; η_p_^2^ = 0.005). Descriptive statistics of the PCS scores are provided in Additional file 1: Table S2.

**Table 3:** Panic disorder, generalized anxiety disorder and the physical component summary (PCS) score (n = 434)

	β (SD)	*p*
*Time effect* 6-month follow-up versus baseline	1.77 (0.73)	0.016*
*Group effect*	–	< .001*
PD versus no PD or GAD	− 6.24 (1.46)	< .001*
GAD versus no PD or GAD	0.64 (1.79)	0.720
PD and GAD versus no PD or GAD	− 5.66 (2.31)	0.015*
PD versus GAD	− 6.89 (2.20)	0.002*
PD and GAD versus PD	− 0.58 (2.64)	0.826
PD and GAD versus GAD	− 6.30 (2.83)	0.027*
*Time* × *group effect*	–	0.585

Adjusted for civil status and family income

SD: Standard deviation; PD: Panic disorder; GAD: Generalized anxiety disorder

^*^p < 0.05

**Fig. 2:**
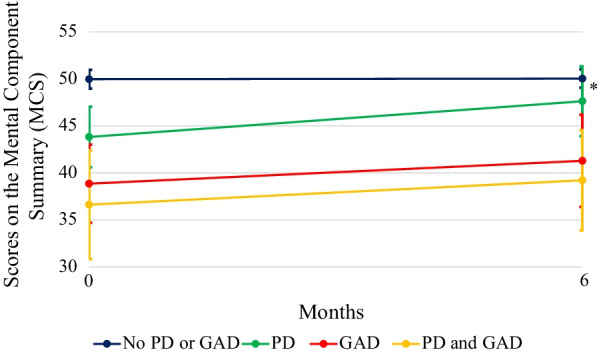
Interaction effect of the Mental Component Summary (MCS) score. *Significant time x group interaction effect after adjustment for differences in civil status

There was a significant group x time interaction for the MCS (*F* (3, 428) = 2.63; *p* = 0.0499 η_p_^2^ = 0.018). At baseline, the patients with no comorbidities had a significantly higher MCS score than those in the three other groups with comorbidities. Furthermore, the patients with GAD, with or without comorbid PD, had a lower MCS score than those with PD and no GAD. At the six-month follow-up, both groups of patients with GAD still had a significantly lower MCS score than those with no comorbidities. They also had a lower MCS score than the patients with PD only, this effect being greater at the follow-up than at baseline. Finally, the patients with PD and those with no comorbidities did not differ significantly from each other at this time point compared to baseline. See Fig. 2 for further details. Descriptive statistics of the MCS scores are provided in Additional file 1: Table 2.

## Discussion

The first objective of this study was to describe and compare NCCP severity in patients with or without comorbid PD or GAD at baseline and at the six-month follow-up. Overall, the patients still reported episodes of NCCP at the six-month follow-up, which corroborates the well-documented persistence of these symptoms in the literature [1, 7, 30, 48]. A time effect of moderate size was found for all the patients (η_p_^2^ = 0.162), as average NCCP severity tended to decrease from baseline to the six-month follow-up, which supports the results of Dammen and colleagues [29]. This improvement in symptom severity could be explained by some form of reassurance obtained during the emergency department visit. Still, average NCCP severity was higher in the patients with comorbid PD or GAD at both time points (η_p_^2^ = 0.029). This result is in line with those indicating that comorbid psychiatric disorders are associated with increased NCCP severity [21, 28–31]. Surprisingly, no significant differences were found between the patients with PD and those with GAD in terms of average NCCP severity. This does not support the primary hypothesis, namely, that patients with PD would report more severe NCCP. The greater-than-expected association of GAD with NCCP severity may be explained by intolerance to uncertainty. The impact of medical uncertainty associated with diagnoses of exclusion, such as NCCP [2, 24], might be underestimated in patients with GAD, who are already well-known for their intolerance to uncertainty. Indeed, intolerance to uncertainty is associated with pain severity through the catastrophizing of pain in patients with chronic pain [49, 50]. Therefore, it is possible that the medical uncertainty associated with NCCP, coupled with the intolerance to uncertainty of patients with GAD, increases anxiety significantly, leading these patients to catastrophic interpretations and an amplified perception of bodily symptoms, as experienced by patients with PD as well. Moreover, tolerance of negative emotional states could also explain the strong association of GAD with NCCP severity. Indeed, tolerance of negative emotional states has been independently associated with chronic pain severity, while being closely linked to avoidance of internal experiences and difficulties in emotion regulation found in patients with GAD [51, 52].

Another surprising result is that the presence of both PD and GAD was not associated with greater NCCP severity than the presence of either disorder. This result is not consistent with that of White and colleagues [21], namely, that patients with more psychiatric disorders report greater NCCP severity. However, this apparent contradiction results may be explained by the inclusion of a greater range of disorders (e.g., all anxiety and mood disorders, substance-related disorders) in that study compared to this one.

The second objective of the present study was to describe and compare health-related quality of life in patients with NCCP, with or without comorbid PD or GAD, at baseline and at six-month follow-up.

Physical quality of life improved over time for all the patients, regardless of the group, which is consistent with previous studies of patients with NCCP with or without PD [8, 29]. This limited improvement (η_p_^2^ = 0.014) could be explained by some form of reassurance obtained by all patients during the emergency department visit. However, one could argue that, in the long term (e.g., one-year or two-years follow-up), certain groups of patients may be at greater risk of negative trajectories than others once the reassurance effect has worn off. Of note, the presence of PD, with or without comorbid GAD, was significantly and meaningfully (η_p_^2^ = 0.260) associated with a diminished physical quality of life over time, which is in line with the primary hypothesis and results of Bull Bringager and colleagues [28]. The results also show a lower physical quality of life in the patients with PD than in those with GAD. This observation corroborates reports that PD is the anxiety disorder that affects the physical functioning subscale the most in patients without NCCP [32, 34].

As regards mental quality of life, a time x group interaction effect of small size (η_p_^2^ = 0.018) was obtained. Indeed, the presence of GAD, alone or in comorbidity with PD, was significantly associated with a lower mental quality of life than PD alone, this effect being greater at the follow-up than at baseline. These results support the primary hypothesis of a greater association of GAD with lowered MCS scores and are also consistent with the positive association found by White and colleagues [21] between the number of disorders and the impairment reported by patients. Moreover, the absence of a significant difference between the patients with PD and those with no comorbidities at the six-month follow-up also suggests that PD has a significant but limited impact on mental quality of life.

In summary, this study highlights the considerable negative impacts of GAD on NCCP and health-related quality of life of patients, which confirms that they represent a particularly vulnerable subgroup of patients, just like patients with NCCP and PD. Therefore, GAD should also be identified early in the process so that patients can benefit from appropriate treatment or referral. Some brief self-report instruments have been shown to be useful in screening for PD in patients with NCCP, such as the Revised Panic Screening Score [53, 54] and the Psychiatric Diagnostic Screening Questionnaire [55, 56]. A similarly brief instrument, such as the GAD-2, could be used to screen for GAD during a patient’s initial visit for NCCP [57]. This study also highlights the differential impact of PD and GAD on health-related quality of life, which reinforces the relevance of assessing specific domains of quality of life because they appear to vary across anxiety disorders. These findings shed light on specific needs of these patients that could be targeted in order to improve their quality of life.

Currently, psychological interventions for NCCP are offered to patients to prevent pain from becoming chronic [58]. While a Cochrane review recommends cognitive-behavioural therapy for these patients, its benefits appear to be modest and largely limited to the first three months after the intervention [58]. As for offering psychological treatment for the comorbid anxiety disorder itself in patients with NCCP, studies show that cognitive-behavioural therapy is effective in reducing PD severity, but little is known about its impact on NCCP [59–61]. With regard to GAD, no study to date has assessed the effectiveness of cognitive-behavioural therapy in patients with NCCP. Based on the findings of the present study, the current intervention for patients with NCCP only is likely to be insufficient to treat patients with NCCP and comorbid PD or GAD.

The primary strengths of this study are its prospective nature, the consecutive sampling, the high retention rate at the six-month follow-up (80.2%) and the large sample size, especially for the assessment of NCCP severity, thanks to the high measure completion rate. Another strength of this study is the application of a standardized psychiatric interview and the high diagnostic reliability achieved. Moreover, the use of generalized linear mixed models helped minimize potential type I errors, as dependence between time points for each patient was considered. Finally, this study was able to address a current knowledge gap by prospectively assessing NCCP severity and health-related quality of life in patients with NCCP and GAD. It also prospectively compared NCCP severity and health-related quality of life according to the patients’ anxiety disorder profile.

This study has some limitations that should be taken into consideration when interpreting the results. First, one should bear in mind that the presence of PD and GAD was not reassessed at follow-up. Considering the chronic course of these disorders over time [31, 62], a potential impact on the results appears unlikely. Second, certain analyses were conducted on small subgroups of patients, and a lack of statistical power could explain some of the negative results. However, group effects were obtained by combining certain subgroups, which made it possible to draw relevant conclusions. Third, potential implications for the findings’ internal validity and generalizability of the results need to be addressed. On one hand, patients who refuse to enrol in the study might have been different from those who participated in the study with respect to some sociodemographic characteristics or general health. On the other hand, significant differences between patients in the final sample and those who were not included in the study should be acknowledged. The patients in the final sample (n = 915) for the first objective (NCCP severity) were more educated, more likely to be married or in a common-law relationship, and had a higher family income. Consequently, one could argue that the patients in the final sample might have had a better understanding of research and better health, which might have resulted in an underestimation of NCCP severity in the present study. The patients in the final sample (n = 434) for the second objective (health-related quality of life) were older and less likely to be working than those who did not complete the measure at either time point. The age difference may mean that the PCS scores were lower than they should have been, while the MCS scores were higher than they should have been [34]. The difference regarding employment status may have led to a lowered health-related quality of life in the study since the patients who were not working might have had poorer health.

## Conclusions

Like PD, GAD is prospectively associated with higher NCCP severity and lowered health-related quality of life. PD appears to be more closely associated with a decrease in the physical component of quality of life, while GAD is more strongly associated with impairment in the mental quality of life. These results highlight the fact that patients with PD or GAD represent a particularly vulnerable subgroup of patients with NCCP. Considering the specific and distinct effect of these disorders on patients could help improve care for this subset of patients with NCCP.

## Supplementary Information

**Additional file 1: Table S1.** Descriptive statistics on average NCCP severity. **Table S2.** Descriptive statistics of the PCS scores.

## Data Availability

The datasets used and analyzed during the current study are available from the corresponding author on reasonable request.

## Abbreviations

ADIS-IV: Anxiety disorders interview schedule fourth edition
ANOVA: Analyses of variance
CISSS-CA: Centre intégré de santé et de services sociaux de Chaudière-Appalaches
GAD: Generalized anxiety disorder
MCS: Mental component summary
NCCP: Noncardiac chest pain
η_p_^2^: Partial eta-squared (effect size)
PD: Panic disorder
PCS: Physical component summary
SD: Standard deviation
SF-12v2: 12-Item short-form health survey version 2
