# Supplementary material for: Anxiety disorders in patients with noncardiac chest pain: association with health-related quality of life and chest pain severity
Source: Health Qual Life Outcomes. 2022 Jan 10;20:7. doi: 10.1186/s12955-021-01912-8 (PMC8751105; doi:10.1186/s12955-021-01912-8)
Supplement: Supplementary file 1 — Additional file 1: Table S1. Descriptive statistics on average NCCP severity. Table S2. Descriptive statistics of the PCS scores. [file 12955_2021_1912_MOESM1_ESM.docx]

Supplementary data

Supplementary Table 1

*Average NCCP severity (N = 915)*

|  | PD  (n = 77) | GAD  (n = 56) | PD and GAD  (n = 48) | No PD or GAD  (n = 734) |
| --- | --- | --- | --- | --- |
| Baseline | 5.32 ± 2.14 | 5.61 ± 2.31 | 5.94 ± 2.14 | 5.07 ± 2.43 |
| Six-month follow-up | 3.31 ± 2.69 | 2.95 ± 2.91 | 3.13 ± 2.68 | 2.20 ± 2.66 |

PD: Panic disorder; GAD: Generalized anxiety disorder.

Supplementary Table 2

*SF-12v2 Physical and Mental Component Summary of health-related quality of life (N = 434)**

|  |  | PD  (n = 38) | GAD  (n = 24) | PD and GAD  (n = 16) | No PD or GAD  (n = 356) |
| --- | --- | --- | --- | --- | --- |
| PCS | Baseline | 38.58 ± 12.15 | 45.82 ± 8.28 | 40.25 ± 8.28 | 46.59 ± 9.09 |
|  | Six-month follow-up | 41.22 ± 11.51 | 48.14 ± 8.83 | 41.36 ± 10.47 | 47.51 ± 9.32 |
| MCS | Baseline | 43.82 ± 9.82 | 38.85 ± 9.88 | 36.61 ± 10.93 | 49.97 ± 9.56 |
|  | Six-month follow-up | 47.62 ± 11.33 | 41.28 ± 11.67 | 39.21 ± 10.05 | 50.03 ± 9.22 |

* A higher score indicates a higher health-related quality of life; PCS: Physical Component Summary; MCS: Mental Component Summary; PD: Panic disorder, GAD: Generalized anxiety disorder.
